# Influence of tumors on protective anti-tumor immunity and the effects of irradiation

**DOI:** 10.3389/fonc.2013.00014

**Published:** 2013-02-01

**Authors:** Gemma A. Foulds, Jürgen Radons, Mira Kreuzer, Gabriele Multhoff, Alan G. Pockley

**Affiliations:** ^1^Department of Oncology, The Medical School, The University of SheffieldSheffield, UK; ^2^Department of Radiation Oncology, Klinikum rechts der Isar, Technische Universität MünchenMunich, Germany; ^3^multimmune GmbHMunich, Germany; ^4^Clinical Cooperation Group - Innate Immunity in Tumor Biology, Helmholtz Zentrum MünchenMunich, Germany; ^5^John van Geest Cancer Research Centre, Nottingham Trent UniversityNottingham, UK

**Keywords:** tumor immunity, T cells, NK cells, tumor microenvironment, immunoregulation

## Abstract

Innate and adaptive immunity plays important roles in the development and progression of cancer and it is becoming apparent that tumors can influence the induction of potentially protective responses in a number of ways. The prevalence of immunoregulatory T cell populations in the circulation and tumors of patients with cancer is increased and the presence of these cells appears to present a major barrier to the induction of tumor immunity. One aspect of tumor-mediated immunoregulation which has received comparatively little attention is that which is directed toward natural killer (NK) cells, although evidence that the phenotype and function of NK cell populations are modified in patients with cancer is accumulating. Although the precise mechanisms underlying these localized and systemic immunoregulatory effects remain unclear, tumor-derived factors appear, in part at least, to be involved. The effects could be manifested by an altered function and/or via an influence on the migratory properties of individual cell subsets. A better insight into endogenous immunoregulatory mechanisms and the capacity of tumors to modify the phenotype and function of innate and adaptive immune cells might assist the development of new immunotherapeutic approaches and improve the management of patients with cancer. This article reviews current knowledge relating to the influence of tumors on protective anti-tumor immunity and considers the potential influence that radiation-induced effects might have on the prevalence, phenotype, and function of innate and adaptive immune cells in patients with cancer.

## Introduction

For many years, the paradigm on which the majority of immunotherapeutic approaches for the treatment of cancer has been based, is that adaptive immune responses to tumors are similar to those that are induced in the generation of immunity to infectious pathogens. Although this is, in part at least, the case, a fundamental difference is that responses induced by infectious pathogens are driven by exogenous (“foreign”) proteins/molecules, whereas those to tumors must be induced by endogenous (“self”) proteins. Although not all tumor antigens are “self” antigens (e.g., EBV antigens, mutated p53), the majority are. For effective immunity, it is therefore necessary to overcome the well-developed capacity of the immune system to regulate responses to self-antigens and tissues.

Tumors can produce immunosuppressive factors such as IL-10, TGF-β, and vascular endothelial growth factor (VEGF). Tumor-derived cytokines such as IL-10 and TGF-β might protect against the development of anti-tumor immunity by influencing the functional capacity of antigen presenting cells (APCs) such as dendritic cells (DCs), and by promoting the generation/differentiation/expansion of immunoregulatory T cell populations which have the capacity to control and prevent immune responses (Ghiringhelli et al., 2005b; Liu et al., 2007; Mahnke et al., 2007a; Biollaz et al., 2009; Conroy et al., 2012; Multhoff and Radons, 2012). The biology of different immunoregulatory T cell populations and the functional significance of tumor-associated immunoregulatory T cells have recently been reviewed (Sakaguchi, 2011; Shevach, 2011; Facciabene et al., 2012; Savage et al., 2013). The net result is a complex relationship between tumors and elements of the protective immune system which has profound influences on the progression and treatment of cancer. It is on the influence of tumors on innate and adaptive immunity by tumors that this article focusses.

## Immunoregulatory T cells

It is now known that thymic deletion of potentially self-reactive T cells cannot explain the lack of immune responsiveness to self-tissues and antigens, as the normal T cell repertoire includes low affinity T cells that are reactive against a number of self-peptides. Mechanisms that are capable of controlling the development of immune responses to self-antigens in the periphery must therefore be present. Over the last few years an anti-inflammatory activity has been shown to segregate, in part at least, into a naturally occurring CD4^+^ T cell subset which constitutively expresses the α chain of the IL-2 receptor (CD25) (Shevach, 2002; Gavin and Rudensky, 2003; Wood and Sakaguchi, 2003; Lee et al., 2004; Waldmann et al., 2004).

In addition to CD25, these cells also express other antigens including the intracellular transcription factor forkhead box p3 (Foxp3) (Sakaguchi, 2004), glucocorticoid-induced TNF receptor family-related gene (GITR), the immunoregulatory antigen CTLA-4, neuropilin-1 (Bruder et al., 2004) and, in the case of humans, low cell surface levels of CD127 (Liu et al., 2006b; Seddiki et al., 2006). The depletion or absence of these cells triggers autoimmune destruction of a variety of tissues (Asano et al., 1996; Suri-Payer et al., 1998; McHugh and Shevach, 2002). Although a multitude of immunoregulatory T and B cell populations have, and are, being discovered and reported upon, CD4^+^CD25^high^ Treg cells are regarded as being intimately involved in the governance of peripheral self-tolerance (Shevach, 2002, 2011; Nelson, 2004; Sakaguchi, 2004, 2011; Facciabene et al., 2012; Savage et al., 2013).

CD4^+^CD25^high^ Treg cells develop in the thymus and represent 5–10% of the peripheral CD4^+^ T cell compartment. Although the expression of Foxp3 is currently accepted as being the most effective marker of Treg cells in mice and humans (Graca, 2005), there is evidence that some Foxp3 negative cells can be suppressive, and Foxp3 is not therefore a definitive marker for Treg cells (Gavin et al., 2007; Curiel, 2007a; Wan and Flavell, 2007). The suppressive effects of naturally-occurring Treg cells are mediated via relatively far-reaching soluble factors and more intimate cell–cell contact, as well as direct cytotoxic effects on effector cell populations (Schmetterer et al., 2012). These cells are also key regulators of anti-tumor immunity (Facciabene et al., 2012; Lindau et al., 2013; Savage et al., 2013).

According to Savage et al. (2013), the biology of tumor-associated Treg cells involves two developmental pathways: (1) the recognition of self-antigen by developing thymocytes within the thymus leads to the development of naturally-occurring Foxp3^+^ Treg (nTreg) cells and (2), naïve CD4^+^ T cells recognize a tumor-associated or tumor-specific antigen at extrathymic sites and, after being activated, develop into an inducible Foxp3^+^ Treg cell (iTreg or Tr1) under a variety of conditions that facilitate/enable tumor immune evasion. These conditions include not only antigen presentation under sub-immunogenic or non-inflammatory conditions, but also chronic inflammation and infections. The observed selective accumulation of Treg cells in the tumor microenvironment suggests that this process can also be driven by tumors.

As hypothesized by Adema and colleagues (Jacobs et al., 2012), four non-mutually exclusive mechanisms can account for the accumulation of Treg cells in the tumor microenvironment: (1) Chemokine secretion induces the selective migration and retention of Treg cells that constitutively express high levels of CCR4 (CCL22, CCL2). (2) Secretion of anti-inflammatory mediators such as TGF-β and indoleamine 2,3-dioxygenase (IDO) converts conventional (naïve) T cells to Treg cells, either directly or via the actions of antigen-presenting cells; (3) A selective survival advantage of Treg cells over other tumor-infiltrating lymphocytes occurs when negative costimulatory signals selectively influence effector T cells (PD-L1, FasL). Treg cells also induce receptor-mediated or cytotoxin-mediated Teff cell depletion; (4) tumor-derived immunosuppressive factors such as IL-10 and TGF-β promote the expansion of nTreg cells and the *de novo* generation of iTreg cells.

The different origins of iTreg cells (non-inflammatory, inflammatory) results in distinct properties of these cells which include differential stabilities (Bilate and Lafaille, 2012). iTreg cells are also generated during homeostasis of the gut and in cancer, although some cancers favor the expansion of nTreg cells. Both pathways converge in the tumor environment and this leads to context-dependent Treg cell functions such as the promotion of metastasis and angiogenesis, as well as the limitation of inflammation and blockage of anti-tumor immunity in response to inflammatory conditions (tissue/organ-specific) and the tumor microenvironment, respectively. The suppressive effect of nTreg cells is mediated via cell contact-dependent mechanisms such as granzyme B/perforin and Fas/FasL (Jonuleit et al., 2001). In contrast, iTreg cells mediate suppression in a cell contact-independent manner (Roncarolo et al., 2006; Bergmann et al., 2008; Mandapathil et al., 2010).

## Immunoregulatory T cells and anti-tumor immunity

As stated above, a wealth of historical and more recent evidence now suggests that CD4^+^CD25^high^ Treg cell populations influence the presence, induction, and maintenance of protective anti-tumor immunity (Raimondi et al., 2007; Facciabene et al., 2012; Lindau et al., 2013; Savage et al., 2013), and their association with the progression of malignant disease has been highlighted by a number of observations (Table 1).

**Table 1:** **Influence of CD4^+^CD25^high^T_reg_ cells on anti-tumor immunity**.

**Observation**	**References**
CD4^+^CD25^high^ Treg cells are potent inhibitors of anti-tumor immune responses and the depletion of Treg cells promotes the rejection of several transplantable murine tumor cell lines including melanoma, fibrosarcoma, leukaemia, and myeloma	Sakaguchi et al., 1995; Onizuka et al., 1999; Shimizu et al., 1999; Steitz et al., 2001; Jones et al., 2002
CD4^+^CD25^high^ Treg cells impair responses to tumor-associated antigens that are expressed as self-antigens	Sutmuller et al., 2001; Golgher et al., 2002
Increased numbers of functionally suppressive CD4^+^CD25^high^ Treg cells are present in the peripheral blood of patients with breast cancer, and also in the tumor microenvironment	Liyanage et al., 2002; Wolf et al., 2003
CD4^+^CD25^high^ T cells from patients with epithelial malignancies are anergic to T cell receptor stimulation and suppress the proliferation of CD4^+^CD25^−^ T cells	Wolf et al., 2003
Using an experimental murine system and CT26 tumor cells, depletion of CD25^high^ Treg cells has been shown to allow the host to induce both CD4^+^ and CD8^+^ anti-tumor responses following tumor challenge. The capacity of the host to mount this anti-tumor response is lost once the number of CD25^high^ Treg cells is restored over time	Casares et al., 2003
The depletion of CD25^high^ Treg cells before immunization with AH1 (a cytotoxic T cell determinant from CT26 tumor cells) permits the induction of a long-lasting anti-tumor immune response which is not observed if immunization is conducted in the presence of CD25^high^ Treg cells	Casares et al., 2003
CD4^+^CD25^high^ Treg cells alone can prevent effective adoptive immunotherapy	Antony et al., 2005
CD4^+^CD25^high^ Treg cells can impair CD8^+^ T cell immunity against tumor/self-antigens	Antony et al., 2005
Depletion of CD4^+^CD25^high^ Treg cells promotes a tumor-specific immune response in mice bearing pancreatic cancers	Viehl et al., 2006
The depletion of CD4^+^CD25^+^Foxp3^high^ Treg cells increases the efficacy of vaccination approaches that are aimed at increasing cellular and humoral immunity to Her-2 which is expressed on primary and metastasized breast cancer cells	Fulton et al., 2006
The proportion of CD4^+^CD25^high^T_reg_ cells is elevated in the peripheral blood of patients with hepatocellular carcinoma (HCC), and their levels positively correlate with tumor burden	Cao et al., 2007
Depletion of CD25^+^ cells results in an accumulation of CD4^+^ and CD8^+^ T cells and NK cells producing IFN-γ in mesothelioma tumor tissue	Rudge et al., 2007
In a syngeneic murine glioma model, combining Treg cell depletion with administration of blocking CTLA-4 mAbs further boosted glioma-specific CD4^+^ and CD8^+^ effector T cells resulting in complete tumor eradication without any signs of autoimmunity. These data illustrate that intratumoral accumulation and activation of CD4^+^FoxP3^+^ Treg cells act as a dominant immune escape mechanism for gliomas	Grauer et al., 2007
The frequency of CD4^+^CD25^high^ Treg increases during disease progression and also following cancer therapy in HNSCC patients with no evident disease compared to untreated patients with active disease	Strauss et al., 2007
CD4^+^CD25^high^T_reg_ secrete IL-10 and TGF-β and mediate immunosuppression in the tumor environment in a cell contact-independent manner	Strauss et al., 2007
Low doses of IL-2 in combination with DC vaccination are able to expand CD4^+^CD25^+^Foxp3^+^ Treg cells in metastatic renal cell carcinoma patients suggesting that a combination of DCS-mediated immunotherapy and Treg depletion may be a promising approach in enhancing the ability of vaccination therapy to elicit effective anti-tumor responses in cancer patients	Berntsen et al., 2010
FOXP3^+^ Treg cells predict poor survival in patients with cyclooxygenase-2–positive uveal melanoma	Mougiakakos et al., 2010
AML and high-risk MDS patients have significantly larger CD4^+^CD25^high^/CD4 and CD4^+^CD25^high^FoxP3^+^/CD4 populations in the periphery compared to patients with autoimmune hematologic diseases and controls, respectively	Moon et al., 2011
Chemotherapy significantly decreased CD4^+^CD25^high^ Treg cell numbers and FOXP3 mRNA expression in advanced esophageal cancer patients	Xu et al., 2011
The frequency of CD4^+^CD25^high^ Treg cells is elevated in HNSCC patients and may be modulated by radiochemotherapy	Schuler et al., 2011
Neoadjuvant sorafenib treatment significantly reduced the percentage of tumor-infiltrating Treg cells in renal cell carcinoma patients	Desar et al., 2011

These cells present a significant barrier to the induction of tumor immunity (Raimondi et al., 2007; Facciabene et al., 2012; Lindau et al., 2013; Savage et al., 2013), and reducing their numbers and/or function is therefore likely to be of therapeutic potential. The evidence that the depletion of CD4^+^CD25^high^ Treg cells enhances the capacity to induce cellular and humoral immunity to Her-2 which is expressed on primary and metastatic breast cancer cells (Fulton et al., 2006) confirms the importance of these cells and highlights the importance of improving our understanding of the influence of the breast tumor microenvironment on protective innate and adaptive anti-tumor immunity.

It is also important to appreciate that other immunoregulatory T cells such as the adaptive or inducible populations (iTreg) are phenotypically and functionally different to the population discussed above and resistant to apoptosis or oncological therapies (Whiteside, 2012). The potent capacity of these cells to suppress effector T cell function involves immunosuppressive cytokines such as TGF-β, IL-10, prostaglandin E_2_, and adenosine that can be produced by solid tumors and/or Treg cells themselves (Erdman et al., 2003; Roncarolo et al., 2006; Bergmann et al., 2008; Mandapathil et al., 2010; Conroy et al., 2012). As mentioned before, differentiation of naïve CD4^+^ T cells into iTreg cells in the periphery is encouraged by tumor antigen in the presence of certain cytokines such as IL-2, IL-10, and TGF-β (Levings et al., 2001b; Bergmann et al., 2007, 2008).

## Influence of tumors and tumor-related factors on immunoregulatory T cell populations

There is now a wealth of evidence indicating that factors present in the tumor microenvironment can foster immune tolerance by generating and inducing the functional capacity of CD4^+^CD25^high^ Treg cell populations (Zou, 2005) and the induction of antigen-specific regulatory T cells from naïve cells (Zhou and Levitsky, 2007). However, the mechanism(s) underlying the recruitment, expansion and/or activation of these cells remain unclear.

The preferential accumulation of functional regulatory T cell populations in tumors could result from an increased recruitment, decreased emigration, and/or the local conversion of naïve T cells to regulatory populations by tumor-derived factors. The conversion of naïve T cells into inducible immunoregulatory T cells involves TGF-β and other factors, and has been reviewed elsewhere (Dons et al., 2012). Should such factors be involved, then their presence might be patient/tumor and/or treatment-specific. Chemokines play a role in migratory events, as tumor cells and microenvironmental macrophages produce the chemokine CCL22, which mediates trafficking of Treg cells to the tumor (Curiel et al., 2004). More recently, it has been shown that tumor hypoxia promotes the recruitment of regulatory T cells to tumors via the induction of the chemokine CCL28 (Facciabene et al., 2011).

A number of tumor-related events could be influential for the induction of regulatory T cell populations. Regulatory T cells can be induced by antigenic stimulation both *in vitro* and *in vivo* [induced regulatory T cells (Bluestone and Abbas, 2003; Vigouroux et al., 2004)], and these can mediate tumor-specific T cell tolerance (Zhou and Levitsky, 2007). Tumors might therefore release antigens and/or other non-antigen-specific factors that activate Treg cells, thereby mediating tumor-related immunoregulation (Antony et al., 2005). It is also possible that factors expressed on, or released from tumors, might promote the development and expansion of CD4^+^CD25^high^ Treg cells.

In this regard, it is known that the prevalence of CD4^+^CD25^high^ T cells in tumor draining lymph nodes and the spleens of mice bearing the pancreatic adenocarcinoma Pan02, increases with tumor growth (Liyanage et al., 2006). Furthermore, tumor-related factors activate CD4^+^CD25^high^ Treg cells (Li et al., 2005), expand CD4^+^CD25^high^ Treg cells and enhance their suppressive capacity (Cao et al., 2007). Gastric cancer cells induce human CD4^+^Foxp3^+^ regulatory T cells via the production of TGF-β (Yuan et al., 2011). It has also been shown that tumor-related factors activate CD4^+^CD25^high^ Treg cells (as indicated by increasing their expression of CD69) (Li et al., 2005), expand CD4^+^CD25^high^ Treg cells and enhance their suppressive capacity (Cao et al., 2007).

It is also possible that the mode of tumor cell death, whether this is induced by normal cell turnover or by therapeutic intervention can influence the qualitative nature and effectiveness of the immune response induced. Cellular necrosis is an inflammatory stimulus, whereas apoptosis can have anti-inflammatory consequences, at least some of which appear to be mediated via the induction of immunoregulatory T cell populations (Groux et al., 1997; Steinbrink et al., 1997, 1999; Lee et al., 1998; Levings et al., 2001a; Yamagiwa et al., 2001).

## Effect of decreasing regulatory T cells on anti-tumor immunity

Modifying the numbers and function of immunoregulatory T cell populations could be of significant therapeutic benefit to patients with cancer (Ménétrier-Caux et al., 2012). One approach which has been considered is the use of DAB(389)IL-2 (also known as denileukin diftitox and ONTAK). This is a recombinant IL-2/diphtheria toxin fusion protein which delivers diphtheria toxin to CD25^+^ cells and thereby abrogates the immunoregulatory influence of CD4^+^CD25^+^ Treg cells (Dannull et al., 2005). Following internalization, protein translation is inhibited and targeted cells undergo apoptosis (Foss, 2000). The administration of ONTAK has been shown to reduce the number of circulating Treg cells and to enhance the magnitude of vaccine-induced, tumor-specific immune responses in patients with renal cell carcinoma (Dannull et al., 2005). It also improves immunity in patients with melanoma (Chesney et al., 2006; Mahnke et al., 2007b). ONTAK has also been shown to decrease the number of circulating CD4^+^CD25^+^ Treg cells and the suppression mediated by these cells in patients with ovarian, lung, breast, and pancreatic cancer (Curiel, 2007b). It also improves immunity and induces tumor regression in a murine model of breast cancer (Knutson et al., 2006).

Daclizumab (Zenapex®) and basiliximab (Simulect®) are anti-human CD25 monoclonal antibodies (mAbs) which have been approved for use in autoimmune disease, transplantation, and cancer, including HTLV-1-induced adult T cell lymphoma/leukemia (reviewed in Ménétrier-Caux et al., 2012). Daclizumab treatment durably reduces circulating CD25^high^FOXP3^+^ Treg cell numbers and promotes the emergence of cancer-specific cytotoxic T cells after vaccination with cancer antigen peptides (hTERT/survivin) in patients with metastatic breast carcinoma (Rech and Vonderheide, 2009). However, one issue which has to be considered using such approaches is the potential to influence the activated effector cells which also express CD25.

It is also possible to block the function of Treg cells via a number of cell surface receptors (reviewed in Ménétrier-Caux et al., 2012). These approaches include the use of the anti-CTLA-4 antagonist mAb, two humanized forms of which are available—BMS (MDX-100: ipilimumab®, Yervov®) and Pfizer (CP675206: tremelimumab®). These have been evaluated in patients with melanoma, renal cell carcinoma, and prostate cancer (amongst others), with response rates of 10–15% (reviewed in Ménétrier-Caux et al., 2012). The observation that ipilimumab® remarkably improved 1- and 2-year survival of patients with Stage IV melanoma in clinical Phase II studies resulted in approval for this indication by the US Food and Drug Administration. However, the use of CTLA-4 blocking agents has been associated with an increased risk of adverse effects including hypophysitis (Blansfield et al., 2005), diarrhea (Wolchok et al., 2010), colitis (Berman et al., 2010), thyroiditis and arthritis (Bronstein et al., 2011), and inflammatory skin rashes (Klein et al., 2009). Recent observations demonstrate the capability of anti-CTLA-4 mAb to enhance the number of Treg cells without affecting overall immune capacity (Maker et al., 2005; Ralph et al., 2010), implies a direct activation of effector T cells by anti-CTLA-4 mAb (Ménétrier-Caux et al., 2012).

Other options include the use of Vascular Endothelial Growth Factor Receptor (VEGFR) antagonists (sutent, sorafenib) (Ozao-Choy et al., 2009; Adotevi et al., 2010). Sorafenib dramatically reduces the number of peripheral and tumor-infiltrating Treg cells in patients with metastatic renal cell carcinoma (Busse et al., 2011; Desar et al., 2011) and sunitinib monotherapy decreases the number of peripheral Treg and has been shown to improve overall survival in >70% of the patients (Adotevi et al., 2010). One can hypothesize that combining CTLA-4 blockage with immunotherapy might improve the overall effectiveness of these approaches. Indeed, ONTAK-mediated elimination of Treg cells followed by vaccination with RNA-transfected DCs significantly improves the stimulation of tumor-specific T cell responses in patients will renal cell carcinoma when compared with vaccination alone (Dannull et al., 2005).

The use of agonistic mAbs against OX40 (CD134), a co-stimulatory molecule of the TNF receptor family (Piconese et al., 2008), represents a further approach to abrogate Treg cell-mediated suppression of anti-tumor immunity (Kitamura et al., 2009) and facilitate tumor rejection (Piconese et al., 2008).

Toll-like receptor (TLR2, TLR8, TLR9) agonists can also be considered as promising tools for blocking Treg cell-mediated immunosuppression. TLRs are involved in the recognition of pathogen-associated molecular patterns (PAMPs) and the activation of processes that lead to innate immune recognition. TLRs are expressed by a range of immune and non-immune cells, including Treg cells, and they play an important role in tumor immunotherapy (van Maren et al., 2008). Pre-treatment of human Treg cells with a mixture of TLR2 ligands (Pam_2_CSK_4_, Pam_3_CSK_4_, and FSL-1) abolishes Treg cell function by down-regulating the Cdk inhibitor p27^Kip1^ and restoring Akt phosphorylation (Oberg et al., 2011). The synthetic bacterial lipoprotein Pam_3_Cys-SK_4_, a TLR1/2 agonist which is capable of modulating T cell immune responses, has been found to induce the expansion of CD4^+^ CD25^+^ Treg cells and CD4^+^ CD25^−^ effector T cells in the absence of APCs (Liu et al., 2006a). Expanded Treg cells showed a transient loss of suppressive activity. Furthermore, Pam_3_Cys-SK_4_ renders effector cells resistant to the suppression of Treg cells by increasing IL-2 secretion. The group of Chu convincingly demonstrated that Pam_3_Cys-SK_4_ treatment of mice with established lung carcinoma, leukemia, and melanoma, respectively, induced tumor regression and a long-lasting protective response against tumor re-challenge (Zhang et al., 2011).

Pam_3_Cys-SK_4_ treatment also reduces the suppressive function of Treg cells and enhances the cytotoxicity of tumor-specific cytotoxic T lymphocytes *in vitro* and *in vivo* (Zhang et al., 2011). Treg cell function can be reversed by synthetic and natural ligands for human TLR8 by a mechanism which is independent of DCs, and the adoptive transfer of TLR8 ligand-stimulated Treg cells into tumor-bearing mice enhances anti-tumor immunity (Peng et al., 2005). Furthermore, a preoperative local administration of the TLR9 agonist CpG B-type oligodeoxynucleotide (ODN) PF-3512676 (formerly known as CPG 7909) lowers the frequency of CD4^+^CD25^high^ Treg cells in the sentinel lymph node of 23 patients with Stage I to III melanoma (Molenkamp et al., 2007).

Another promising immunotherapy involves blockage of PD-1. Programmed death 1 (PD-1, CD279) is a key immune-checkpoint receptor expressed by several T cell subsets including Treg cells which plays an important role in the balance and regulation of adaptive immune responses. Programmed death ligand 1 (PD-L1) is constitutively expressed by B cells, DCs, macrophages, and T cells and can also be found on different tumor cells of human cancer (Jacobs et al., 2009). Activation-induced upregulation of PD-L1 occurs via TLR4 and STAT1 signaling (Loke and Allison, 2003; Freeman et al., 2006). Inhibition of PD-1 and PD-L1 interactions enhances T cell responses *in vitro* and mediates anti-tumor activity in pre-clinical models (Topalian et al., 2012a). Upregulation of PD-1 and its ligand might therefore be associated with immune evasion and inhibition in tumor-bearing hosts. Levels of T cells expressing PD-1 are upregulated in patients with high-risk renal cell carcinoma and patients with PD-1-positive T cells are at a significantly higher risk of cancer-specific death compared with patients harboring low PD-1-expressing T cells (Thompson et al., 2007). The blockage of the PD-1/PD-L1 pathway using anti-PD-L1 mAbs abrogates Treg cell-mediated immune regulation *in vitro* and tolerance induction *in vivo* in mice (Kitazawa et al., 2007). Treg cell and PD-1 pathway signals have been studied in tumor-bearing patients. The function and phenotype of Treg cells and the expression of PD-1 and PD-L1 on different cell populations from the peripheral blood of patients with high-risk-resected stage III and IV melanoma has been studied, and PD-1 blockage found to augment the generation of melanoma antigen-specific cytotoxic T cells by stimulating their proliferation and, indirectly, by masking their suppression by Treg cells (Wang et al., 2009). PD-1 blockage of Treg cells also diminished their inhibitory function (Wang et al., 2009).

In a dose-escalation study, the anti-PD-1 mAb BMS-936558 (also termed MDX-1106 and ONO-4538) has been used as single-agent in patients with advanced solid refractory tumors (Brahmer et al., 2010). This Phase I study demonstrated a favorable safety profile and preliminary evidence of clinical activity, thereby establishing the basis for a multiple-dose Phase I trial (Topalian et al., 2012b). PD-1 blockage extended the spectrum of clinical activity by immunotherapy beyond immunogenic tumor types, such as melanoma and renal-cell cancer, to treatment-refractory, metastatic non-small-cell lung cancer that is commonly not considered as being responsive to immunotherapy. This study and a companion study with anti-PD-L1 antibody (Brahmer et al., 2012) describe clinical activity with these agents validating the impact of the PD-1/PD-L1 pathway for the treatment of certain cancers. Phase II trials are under way (ClinicalTrials.gov numbers, NCT01354431 and NCT01358721), and Phase III studies with anti-PD-1 antibody for the treatment of non-small-cell lung cancer, melanoma, and renal cell cancer are being designed. Such treatment regimens offer a promising therapeutic approach for other tumor entities.

Given the difficulties that are associated with specifically targeting regulatory T cells, interest in the use of cyclophosphamide for inhibiting regulatory T cells and enhancing the induction of anti-tumor immune responses has developed (Le and Jaffee, 2012). A current perspective on the potential use of cyclophosphamide for reducing/eliminating the negative impact of regulatory T cells on protective anti-tumor immunity and thereby enhancing the efficacy of immunotherapeutic strategies has been provided elsewhere (Le and Jaffee, 2012). The key elements of the approach are that low-dose cyclophosphamide can enhance tumor-specific immune responses, despite the fact that it transiently decreases the frequency of regulatory T cell populations (Machiels et al., 2001; Motoyoshi et al., 2006; Emens et al., 2009) and that a metronomic (iterative, low-dose) approach results in a more prolonged suppression of regulatory T cells which returns to baseline within 4–6 weeks, even in the presence of continued administration (Cerullo et al., 2011; Le and Jaffee, 2012). It is also interesting to note that the downstream effects of cyclophosphamide treatment include the appearance of high avidity T effector cells (Ercolini et al., 2005; Laheru et al., 2008; Le and Jaffee, 2012).

It is important to appreciate that the influence of regulatory T cell populations on the induction of protective immunity might extend beyond their effects on adaptive T cell immunity, as CD4^+^CD25^high^ Treg cells also inhibit the cytotoxic activity of freshly isolated natural killer (NK) cells via their production of TGF-β (Ghiringhelli et al., 2005a). CD4^+^CD25^high^ Treg cells from cancer patients effectively inhibit NK cell-mediated cytotoxicity (Wolf et al., 2003) and the depletion of CD4^+^CD25^+^ Treg cells enhances NKT cell-mediated anti-tumor immunity in a murine mammary breast cancer model (Hong et al., 2010). It is also apparent that the relationship between Treg cells and NK cells is reciprocal, as NK-dependent increases in CCL22 secretion selectively recruits Treg cells to the tumor microenvironment (Mailloux and Young, 2009).

Given the apparent ability of the tumor microenvironment to foster immune tolerance by generating and inducing the functional capacity of regulatory T cell populations (Zou, 2005; Whiteside, 2012), it is essential that we better understand the influence that the tumor microenvironment and treatment modalities have on the induction and progression of the protective anti-tumor immunity which is mediated by T cells and NK cells.

## MHC class I expression, NK cells, and tumor immunity

Approximately 40–90% of human tumors derived from various MHC class I positive tissues are reported to be MHC class I deficient, and MHC class I downregulation is an important mechanism of tumor escape from T cell-mediated immune responses (Garrido et al., 1993; Restifo, 1996; Algarra et al., 1997; Zheng et al., 1999; Johnsen et al., 2001; Groth et al., 2011). Decreased or absent MHC class I expression is frequently associated with the invasive and metastatic tumor phenotype (Garrido and Algarra, 2001; Bubenik, 2003).

Although the modulation of MHC class I expression has a significant potential impact on the application of T cell-based immunotherapies (Marincola et al., 2000), it does render tumor cells to be more susceptible to NK cells which target MHC class I negative (missing self) cells (Kärre et al., 1986; Ljunggren and Kärre, 1990). Proof for the receptor inhibition model of the “missing self” hypothesis comes from the work of Karlhofer and Moretta who identified MHC I-specific inhibitory receptors on the surface of NK cells such as human p58 (later termed KIR2DL; Moretta et al., 1993), mouse-specific inhibitory Ly-49 receptors (Karlhofer et al., 1992) and the multi-species heterodimeric receptor complex CD94/NKG2D (Aramburu et al., 1990; Carretero et al., 1997).

NK cells provide an essential defence against this response and their importance is illustrated by the observation that downregulation of HLA class I is associated with an improved survival of patients with non-small cell lung carcinoma (Ramnath et al., 2006), uveal melanoma (Jager et al., 2002), breast carcinoma, (Madjd et al., 2005) and colon cancer (Menon et al., 2002).

NK cells are large granular innate immune cells which account for 5–20% of human lymphocytes and can spontaneously recognize virally-infected and cancer cells (Kiessling et al., 1975; Langers et al., 2012). NK cells are characterized by the absence of the characteristic T cell antigens CD3 and CD4 (Stobo et al., 1973). Based on their expression of CD56 (Neuronal Cell Adhesion Molecule) and CD16 (Fcγ receptor III; involved in antibody-dependent cellular cytotoxicity, ADCC) peripheral blood NK cells are grouped into two populations by the literature. The predominant population (90%) of NK cells comprises CD56^low^CD16^high^ cells which express perforin and granzymes, and exert cytotoxic functions including ADCC. CD56^high^CD16^−^ NK cells are mainly found in lymph nodes and comprise only 5% of the NK cells in peripheral blood. Stimulated by macrophage and DC-derived type I interferons (IFNs), IL-2 or IL-15, CD56^high^CD16^−^ NK cells are the primary producers of IFN-γ and promote the activation of immune effector cells. An additional population of CD56^high^CD16^+^ cells which accounts for 5% of peripheral blood NK cells exhibit unknown functions (Nagler et al., 1989; Beziat et al., 2011). However, they have been suggested to be an intermediate in the maturation from CD56^high^CD16^−^ to CD56^low^CD16^+^ (Beziat et al., 2011).

NK cells express a large profile of inhibitory and activating receptors, the latter also comprising receptors for cytokines, chemokines, and adhesion molecules (Table 2), and NK cell functional activity depends on the balance of the signals that are delivered (Vivier et al., 2011). The intracellular domains of activating receptors consist of immunoreceptor tyrosine-based activation motives (ITAMs) or DAP10 with its transmembranic aspartic acid residues, and these receptors include NKG2D and CD16 (Vivier et al., 2004).

**Table 2:** **NK cell receptors**.

**Activating receptors**	**Inhibitory receptors**	**Chemotatic receptors**	**Cytokine receptors**	**Adhesion receptors**
mAct.Ly49	CEACAM-1	CCR2	IL-1R	CD2
2B4	CD94/NKG2A	CCR5	IL-2R	DNAM-1
CD16	mInh.Ly49	CCR7	IL-12R	β1 integrins
CD84	hKIR-L	CXCR1	IL-15R	β2 integrins
CD94/NKG2C	KLRG-1	CXCR3	IL-18R	
CRACC	LAIR-1	CXCR4	IL-21R	
hKIR-S	hLILRB1	CXCR6	IFNAR	
Ly9	mNKR-P1B	CX3CR1		
NKG2D	mNKR-P1D	hChem23R		
mNKG2D-S	TIGIT	S1P5		
NKp46				
hNKp30				
hNKp44				
hNKp80				
mNKR-P1C				
NTBA				

NK cells use a wide array of activating and inhibitory receptors which recognize specific ligands expressed by target cells. MHC class I molecules expressed on self cells are engaged by NK cell inhibitory receptors such as Ly49 in mice (mInh.Ly49) and killer immunoglobulin-like receptors (KIR) in humans (h). In contrast, expression of stress or pathogen-induced ligands, downregulation of MHC class I on target cells as well as transformation-mediated ligand expression are recognized by NK cell activating receptors (Table adapted from Vivier et al., 2011, Science 331, 44–49).

NKG2D belongs to the C-type lectin like family and recognizes stress-inducible ligands such as MICA/B or ULBPs (Martinović et al., 2011) and CD16 is the main inducer of ADCC. Receptors that are characteristic of NK cells include natural cytotoxicity receptors (NCRs) such as NKp46 which exert activating functions. Inhibitory receptors are monomeric, associated with immunoreceptor tyrosine-based inhibiting motifs (ITIMs) and exert their function via the recognition of MHC class I and related molecules (Vivier et al., 2004). One inhibitory receptor which recognizes HLA-E either alone or in complex with CD94 is NKG2A (Langers et al., 2012).

Receptors of the killer-immunoglobulin (Ig) like receptor family (KIRs) possess two or three extracellular Ig-like domains and integrate incoming activating or inhibitory signals, but recognize MHC class I molecules with higher affinity than ligands of activating KIRs (Martinović et al., 2011). Autoimmunity is prevented by the low expression of stress-induced ligands and the high expression of MHC class I molecules (Vivier et al., 2008; Langers et al., 2012).

## NK cells and the tumor microenvironment

One way via which tumors might avoid oncolysis is by altering NK cell surface receptors. The expression of inhibitory receptors NKG2A and CD85 on NK cells is upregulated in patients with breast cancer and melanoma (Mamessier et al., 2011a,b). Furthermore, the expression of activating receptors including NKG2D, DNAM-1, Nkp30, and CD16 is downregulated in patients with invasive breast cancer and metastasis to distant sites, with the poorest prognosis being linked to the downregulation of almost all activating receptors (Mamessier et al., 2011a,b; Martinović et al., 2011).

NKG2D expression is reduced in patients with gastric cancer and this is likely to have clinical relevance given that NK cells in patients with lymph node metastases exhibit lower NKG2D expression than those patients with no metastases (Saito et al., 2012). The same study demonstrated a lower expression of NKG2D on NK cells within the primary tumor than their circulating counterparts, and also that NKG2D expression increased after surgery (Saito et al., 2012). Expression of the activation receptors NKp30, NKp46, and NKG2D, but not NKp80 and 2B4 have been reported to be reduced in patients with cervical cancer and with precursor lesions when compared to healthy controls (Garcia-Iglesias et al., 2009). *In vitro* studies have demonstrated that factors released by cervical cancer cell lines significantly reduce NKG2D expression on NK cells and their cytotoxic activity (Jimenez-Perez et al., 2012) and that ovarian and cervical cancer cell lines expressing CD155 can downregulate the expression of the DNAX accessary molecule 1 DNAM-1 on NK cells (Carlsten et al., 2009).

It has recently been shown that the expression of tumor necrosis factor superfamily ligands (TNFSFLs) by NK cells and apoptotic tumor activity are suppressed in patients with head and neck cancer. This suppression is tumor-dependent and possibly mediated by soluble TNF superfamily receptors (solTNFSFRs) (Baskic et al., 2012).

NK cell activity can also be modified by increasing the expression of inhibitory receptor ligands. Ovarian and cervical cancers have been reported to exhibit an upregulated expression of HLA-E, a ligand for the inhibitory CD94/NKG2A complex and the upregulation of HLA-E and the infiltration of cytolytic T cell populations could be neutralized by strong overexpression of the NKG2A ligand (Gooden et al., 2011). Expression of the inhibitory receptor CD158b and the proportion of NK cells expressing it are increased in patients with non-small cellular lung cancer (Al Omar et al., 2011).

With regards to NK cell function, the tumor microenvironment and/or factors derived therefrom have been found to impair NK cell-mediated anti-tumor protection. NK cell numbers can be reduced, as has been found in patients with non-small cellular lung cancer (NSCLC) and melanoma (Al Omar et al., 2011; Martinović et al., 2011), and this might impact on the overall potential of patients to elicit NK cell mediated immunity. Degranulation and NK cell-mediated killing, as well as IFN-γ and TNF-α secretion and induction of ADCC is impaired in patients with metastatic breast cancer via a mechanism which appears to involve TGFβ-1 and prostaglandin E_2_ (PGE_2_) (Holt et al., 2011; Mamessier et al., 2011a,b). These findings have been confirmed by studies that have related an NK cell functional profile with clinical stages in melanoma and demonstrated that CD107a (a marker for degranulation), IFN-γ and TNF-α levels as well as NKG2D were downregulated, whereas CD158b, an inhibitory receptor was upregulated (Martinović et al., 2011). An interesting finding has been that NK activity in the peripheral blood of patients with breast cancer is lower than that in controls and also that the activity of NK cells in patients with HER2- cancers is significantly lower than that in patients with HER2+ tumors (Dewan et al., 2009). NK cell activity has also been shown to be reduced in patients with cervical cancer (Garcia-Iglesias et al., 2009).

Tumors and macrophages produce H_2_O_2_ which can be detected by flow cytometry using intracellular formation of 2′,7′-dichlorodihydrofluorescein as a measure of reactive oxygen species. There is a negative correlation between CD56^dim^ cell numbers and H_2_O_2_ concentration in gastric and oesophageal cancer (Izawa et al., 2011). This population is more susceptible to apoptosis than CD56^bright^, and an observed impairment of ADCC could be reversed by catalase (Izawa et al., 2011). PGE_2_ secretion depends on the rate limiting enzyme cyclooxygenase-2 (COX-2) which is overexpressed in some cancers. PGE_2_ also has a negative influence on NK cell function via the receptors CD16, NCRs, and NKG2D (Holt et al., 2011).

However, it remains unclear whether the impaired functionality of patient-derived NK cells is permissive of the development of cancer or whether the observed altered phenotype and function result from its presence.

## Effect of radiotherapy on a tumor's capacity to influence protective innate and adaptive immunity

The direct effect of irradiation on immune cells and its influence of protective anti-tumor immunity has been considered elsewhere in this Research Topic (Manda et al., 2012; Multhoff and Radons, 2012; Rödel et al., 2012; Rubner et al., 2012; Schmid and Multhoff, 2012). However, there is much less information relating to the influence of radiotherapy on the tumor's capacity to modify anti-tumor immunity, particularly with regards to direct effects of tumor-derived factors on infiltrating NK and immunoregulatory T cell populations. In contrast to the immunosuppressive effects of whole body irradiation, focal radiation such as that used for treatment of many types of solid tumors has the capacity to influence the tumor microenvironment in a way which can enhance the infiltration and the activation of immune cell types which might foster and/or suppress tumor development (de Visser et al., 2006; Shiao and Coussens, 2010).

Radiotherapy induces the expression of nuclear factor (NF)-κB and this has a number of downstream effects with regards to the expression of molecules that promote a pro-inflammatory environment. Radiotherapy stimulates the migration and function of leukocytes via the release of cytokines such as TNF-α (Shakhov et al., 1990), IL-1 (Mori and Prager, 1996), chemokines (Wickremasinghe et al., 2004), the expression of adhesion molecules such as ICAM-1 and E-selectin on vascular endothelial cells within the tumor microenvironment (Iademarco et al., 1992; Caldenhoven et al., 1994; Schindler and Baichwal, 1994; Hallahan et al., 1996; Handschel et al., 1999). *In vivo* experiments using a murine model of mammary carcinoma have demonstrated that radiotherapy-induced expression of the chemokine CXCL16 is an important mechanism which mediates the infiltration of CD8^+^ T effector cells following treatment, as the recruitment of CD8^+^ T cells and responsiveness to treatment are reduced in mice that are deficient for its ligand (CXCR6) (Matsumura et al., 2008).

*In vitro* experiments using a range of different tumor cell types also suggest that the induction of CXCL16 is a common response to radiotherapy (Matsumura and Demaria, 2010). This could have far-reaching effects with regards to the efficacy of radiotherapy and the immunological mechanisms that are involved. Stromal cell-derived factor (SDF)-1α also appears to an important factor for to the immunological consequences of radiotherapy, as inhibiting its pathway prevents macrophage infiltration and delays tumor regrowth (Kozin et al., 2010).

Ionizing radiation also impacts signaling via pattern recognition receptors (PRRs). These receptors interact with exogenous PAMPs such as endotoxin and endogenous “alarmins” such as high-mobility-group-box 1 (HMGB1), hyaluronan, and heat shock (stress) proteins which together comprise the group of danger-associated molecular patterns (DAMPs) (Bianchi, 2007; Kawai and Akira, 2011). The export of DAMPs is achieved by several mechanisms such as (1) leakage from necrotic cells, (2) increased synthesis and post-translational modification in response to inflammation, and (3) degradation of inactive precursors into TLR-mimetic cleavage products in inflammatory environments (Mencin et al., 2009).

The impact of PRR signaling on radiation responses has been documented by Shan and colleagues who demonstrated that radiation-induced release of IL-12 and IL-18 from macrophages is accompanied by NF-κB activation and an upregulation of CD14 and TLR4/MD2 expression, thereby implying the involvement of the Toll signaling pathway (Shan et al., 2007). On B cells and DCs, TLR-related radioprotective 105 kDa (RP105) was identified as being similar to TLR4 because of its ability to interact with the MD2-like adaptor MD1 (Miyake et al., 1995; Fugier-Vivier et al., 1997). RP105/MD1 directly interacts with TLR4/MD2, thus abolishing the LPS binding capacity of the complex (Divanovic et al., 2005). Together with its ability to regulate TLR4 signaling *in vitro* and LPS responses *in vivo*, RP105 can be considered as being a negative regulator of TLR4 responses.

From these observations it can be assumed that pro-inflammatory responses to radiation and TLR signaling not only increase the impact of tumor-promoting factors in the tumor and the microenvironment, but also might function as crucial targets in radiotherapy. It is interesting to note that the radiation-induced release of HMGB1 by dying tumor cells enables the manifestation of tumor antigen-specific T cell immunity (Apetoh et al., 2007). This pathway depends on the interaction of HMGB1 with TLR4 expressed on DCs. During chemotherapy or radiotherapy, DCs require signaling through TLR4 for efficient processing and cross-presentation of antigen from dying tumor cells. Moreover, patients with breast cancer who carry a TLR4 “loss-of-function” allele relapse more quickly after radiotherapy and chemotherapy than those carrying the normal TLR4 allele.

These results delineate a clinically-relevant immunoadjuvant pathway which is triggered by tumor cell death. Silencing of HMGB1 expression by an HMGB1-specific RNAi lentiviral vector has been shown to reduce matrix metalloproteinase 9 (MMP9) expression and metastatic capacity in MGC-803 gastric carcinoma cells (Song et al., 2011). HMGB1-specific silencing also significantly decreased cell proliferation and sensitized cells to oxaliplatin-induced apoptosis mediated via the caspase-3 pathway (Song et al., 2011), rendering HMGB1 a promising target structure in cancer therapy.

An intriguing novel observation has reported by Kono and colleagues who demonstrated that 38% of patients with oesophageal squamous cell carcinoma harbored elevated HMGB1 serum levels after chemoradiotherapy and showed concomitant tumor antigen-specific T cell responses (Suzuki et al., 2012). The same study revealed an upregulated HMGB1 expression within the tumor microenvironment in patients with ESCC after preoperative chemoradiotherapy, but not in those without chemoradiotherapy, and the degree of HMGB1 positively correlated with patient survival (Suzuki et al., 2012). Both, irradiation and chemotherapy induces upregulation of HMGB1 and the chaperone protein calreticulin. Furthermore, HMGB1 is able to induce maturation of DCs, implying that chemoradiation induces tumor antigen-specific T cell responses, and that chemoradiation-mediated HMGB1 production is related to clinical outcome (Suzuki et al., 2012).

Using an *in vitro* approach, in which the transmigration of T cell populations toward supernatants derived from primary cultures of tumor cells derived from patients with head and neck carcinomas, Schmidtner et al. (2009) have demonstrated that supernatants from irradiated cells significantly decrease the transmigration of CD4^+^CD25^high^Foxp3^+^ Treg cells, yet had no effect on the transmigration of CD4^+^CD25^−^ T cells. The observed effects on cell migratory properties have been attributed to treatment-associated increases chemokine (C-C motif) ligand 22 (CCL22) levels in the tumor cell supernatants (Schmidtner et al., 2009). These findings contrast with the effects that are seen following hyperthermia treatment which appears to promote the migration of CD4^+^CD25^high^Foxp3^+^ Treg cells (Schmidtner et al., 2009). These potentially important findings indicate that radiotherapy may play some role in reducing the capacity of tumors to promote the prevalence of CD4^+^CD25^high^Foxp3^+^ Treg cells in the tumor microenvironment and might, as a consequence, facilitate the induction or protective innate and adaptive tumor immunity.

In an attempt to increase the therapeutic benefit of irradiation, several studies have combined molecular oncology therapeutics with radiation. Radiation sensitization, in which cytotoxic enhancers co-operate with radiation within the radiation field, aims to produce a greater (synergistic) anti-tumor effect than would be expected from simple additive cell killing (Zaidi et al., 2009). Oncolytic viruses represent prime candidates for enhancing the immunogenicity of the tumor microenvironment. Oncolytic virotherapy may be immunomodulatory via tumor cell death, production of endogenous danger signals, the release of tumor-derived cytokines and direct effects upon cells of the innate immune system (Prestwich et al., 2008).

Pre-clinical models suggest that tumor viral therapy mediates an early influx of immune cells, such as macrophages and NK cells (Benencia et al., 2005; Diaz et al., 2007). These changes within the tumor hold the potential to alter the pre-existing immunosuppressive microenvironment, in favor of the generation of therapeutic immune responses. DCs are critical for the subsequent generation of antigen-specific or adaptive immune responses. According to Prestwich et al. (2008), the outcome of the innate response is finely balanced between promotion of tumor clearance and viral clearance that limits the efficacy. Strategies that involve combining oncolytic virotherapy with external beam radiotherapy may help to exploit synergies between the two treatment modalities (Hingorani et al., 2007; Harrington et al., 2008).

Melcher and colleagues have demonstrated a synergy between oncolytic reovirus RT3D, a naturally occurring nonpathogenic, double-stranded RNA virus isolated from the respiratory and gastrointestinal tracts of humans and external beam radiotherapy in tumor cell lines *in vitro* and in three different *in vivo* tumor models (Twigger et al., 2008). The same group has now completed a Phase I dose-escalation study of this combination strategy in patients receiving two different dose schedules of palliative radiotherapy and confirmed the safety and tolerability of this approach (Harrington et al., 2010). The study further showed that virus is not shed after administration, thereby opening the way for outpatient treatment regimens. Most importantly, the ease of virus administration and the fact that there was no exacerbation of radiation-induced toxicity strongly support development of this combinatory treatment in patients with newly diagnosed, radiocurable cancers.

Several potentially positive theoretical interactions exist between RT3D and radiotherapy. Tumor radiation resistance is, at least partly, mediated by the Ras signaling pathway (McKenna et al., 2003). Moreover, activating Ras mutations, EGFR overexpression, and phosphorylation of Akt and phosphoinositide-3-kinase have been found as being associated with radioresistance *in vitro* and, with respect to EGFR and Akt, to the failure of radiotherapy in cancer patients (Gupta et al., 2002, 2003; McKenna et al., 2003). Blockage of the Ras signaling pathway sensitizes cells to radiation-induced cytotoxicity (Bernhard et al., 1996; Russell et al., 1999).

Radiotherapy in combination with oncolytic reovirus also represents a promising immunotherapeutic approach, as radiotherapy enhances T cell trafficking (Lugade et al., 2005), antigen presentation, and T cell recognition of tumor cells (Reits et al., 2006). Radiotherapy is also locally immunosuppressive by killing lymphocytes, and the optimal combination to enhance anti-tumor immune responses will require careful consideration of dose fractionation and treatment scheduling.

Another potentially important element of the tumor microenvironment which might be altered following the induction of cell death following radiotherapy relates to the release of heat shock proteins. Although typically regarded as being intracellular proteins, stress proteins, including members of the 60 and 70 kDa families (Hsp60, Hsp70) can be released from a variety of cell types and have been identified in the peripheral circulation in a number of healthy and diseased states (Pockley et al., 1998, 1999, 2000; Rea et al., 2001; Pockley, 2003; Henderson and Pockley, 2010; Henderson et al., 2010). In addition to acting as potent inducers of inflammatory immunity, these proteins can also ameliorate inflammatory events/conditions by inducing/activating regulatory T cell populations (Borges et al., 2012) or by reducing T cell responses and the stimulatory capacity of monocyte-derived DCs (Stocki et al., 2012).

Hsp70 can be released from human glioma, prostate cancer cell lines, the human erythroleukemic cell line K562, and the 4T1 breast carcinoma cell line (Guzhova et al., 2001; Wang et al., 2004; Bausero et al., 2005; Evdonin et al., 2006; Mambula and Calderwood, 2006a,b). Although the relationship between intracellular Hsp70 expression and release has yet to be clarified, increasing the expression of Hsp70 by transfecting prostate cancer cells with cDNA encoding for human Hsp70 enhances their release of Hsp70 (Wang et al., 2004), as does heat treatment (Mambula and Calderwood, 2006a). Non-lethal heat, IFN-γ, and IL-10 also increase Hsp70 release from K562 and 4T1 cells (Bausero et al., 2005). Although in a small cohort of patients, it has been reported that radiotherapy increases circulating Hsp70 levels in patients with prostate cancer and it might be that this reflects increases in Hsp70 levels within the tumor microenvironment (Hurwitz et al., 2010).

Although one study reports that Hsp70 release from prostate cancer cells protects against tumor growth (Wang et al., 2004), presumably via its capacity to induce tumor-specific immunity (Calderwood et al., 2005, 2006), Hsp70 might also adversely influence anti-tumor immunity by activating CD4^+^CD25^high^ Treg cells via its capacity to interact with TLRs expressed thereon. Released Hsp70 might also have direct effects on tumor cell survival following radiotherapy, as radiation-induced tumor cell killing has been shown to be significantly enhanced by the addition of Hsp70 protein via a mechanism which appears to involve necrosis rather than apoptosis (Schilling et al., 2009).

**Figure 1:**
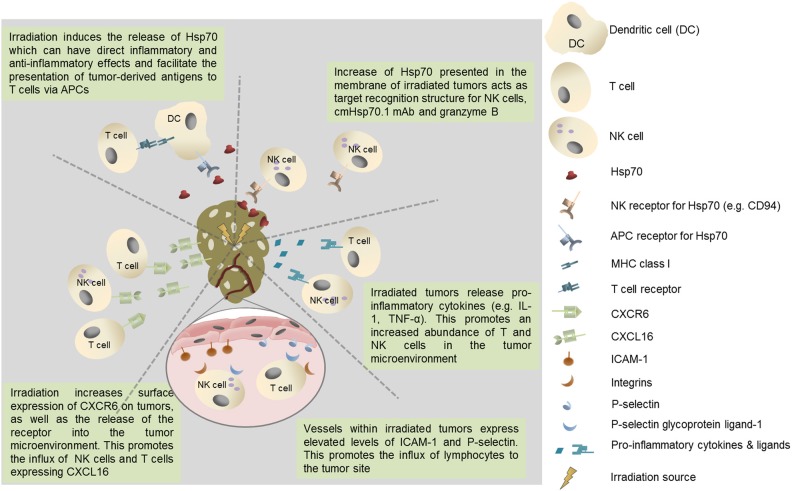
**Potential immunological benefits of tumor irradiation.** Increased recruitment of immunoregulatory T cell populations could negatively impact on protective anti-tumor immunity.

In addition to the secretion of Hsp70 from viable tumor cells and its inevitable release from necrotic cells within the tumor mass, a form of Hsp70 is frequently expressed on the membranes of a number of cancers, and metastases derived therefrom, but not on their non-malignant counterparts (Multhoff et al., 1995, 1997; Botzler et al., 1996, 1998; Multhoff and Hightower, 1996; Multhoff et al., 2001; Gehrmann et al., 2002; Shin et al., 2003; Multhoff, 2007, 2009). It is also the case that the expression of this membrane form of Hsp70 is enhanced by clinically applied interventions such as radio- and chemotherapy (Gehrmann et al., 2008a,b, 2010) (Figure 1).

Membrane Hsp70 acts as a tumor-specific recognition structure for CD94^+^ NK cells and, in the presence of Hsp70 and cytokine (IL-2/IL-15) co-activation, it enhances the capacity of NK cells these cells to kill membrane Hsp70 positive tumor cells (Botzler et al., 1996, 1998; Gross et al., 2003a,b). Membrane Hsp70 expressing tumors can also be imaged and targeted using a specific monoclonal antibody (cmHsp70.1) or a glycosylated recombinant human granzyme B (Stangl et al., 2010, 2011; Gehrmann et al., 2011, 2012). It might therefore be that the release of heat shock proteins such as Hsp70 into the tumor microenvironment by radiation-induced cell death has a number of immunoregulatory effects that influence many aspects of protective anti-tumor immunity (Figure 1).

## Concluding statement

Although it is known that tumors can have a range of immunoregulatory effects that can have tumor-suppressive and tumor-stimulatory properties, an improved insight into the mechanisms and factors involved is required in order to design strategies for promoting the efficacy of therapeutic interventions such as radiotherapy. Focal radiotherapy can elicit anti-tumor immunity by the following mechanisms: (1) boosting trafficking of APCs to the tumor site, (2) augmenting antigen uptake of irradiated tumor cells; (3) increasing the maturation of APCs, (4) inducing maturation of immune effector cells in order to generate a robust immune response, and (5) limiting the immunomodulatory capacity of Treg cell populations.

Accumulation of Treg cells in cancer patients is a major factor in tumor immune escape. Targeting of these cells might thus provide a mechanism by which anti-tumor immune responses could be restored. Immunotherapeutic strategies which target Treg cells and deplete functional Treg cells from the tumor microenvironment cells appear to shift the immune balance in support of effective anti-cancer immune responses. It is likely that effective anti-cancer strategies will depend on multimodal approaches which combine immunotherapy with targeted therapies that stimulate protective immune effector activity and downregulate the suppressive effects of Treg cells.

### Conflict of interest statement

The authors declare that the research was conducted in the absence of any commercial or financial relationships that could be construed as a potential conflict of interest.
